# Effects of PIN on Osteoblast Differentiation and Matrix Mineralization through Runt-Related Transcription Factor

**DOI:** 10.3390/ijms21249579

**Published:** 2020-12-16

**Authors:** Kyung-Ran Park, SooHyun Kim, MyoungLae Cho, Sang Wook Kang, Hyung-Mun Yun

**Affiliations:** 1 Department of Oral and Maxillofacial Pathology, School of Dentistry, Kyung Hee University, Seoul 02447, Korea; rudfks282@khu.ac.kr; 2 National Institute for Korean Medicine Development, Gyeongsan 38540, Korea; beluga81@nikom.or.kr (S.K.); meanglae@nikom.or.kr (M.C.)

**Keywords:** *Styrax Japonica* Sieb. et Zucc, PIN, osteoblast, migration, differentiation, mineralization

## Abstract

*Styrax Japonica* Sieb. et Zucc. has been used as traditional medicine in inflammatory diseases, and isolated compounds have shown pharmacological activities. Pinoresinol glucoside (PIN) belonging to lignins was isolated from the stem bark of *S. Japonica.* This study aimed to investigate the biological function and mechanisms of PIN on cell migration, osteoblast differentiation, and matrix mineralization. Herein, we investigated the effects of PIN in MC3T3-E1 pre-osteoblasts, which are widely used for studying osteoblast behavior in in vitro cell systems. At concentrations ranging from 0.1 to 100 μM, PIN had no cell toxicity in pre-osteoblasts. Pre-osteoblasts induced osteoblast differentiation, and the treatment of PIN (10 and 30 μM) promoted the cell migration rate in a dose-dependent manner. At concentrations of 10 and 30 μM, PIN elevated early osteoblast differentiation in a dose-dependent manner, as indicated by increases in alkaline phosphatase (ALP) staining and activity. Subsequently, PIN also increased the formation of mineralized nodules in a dose-dependent manner, as indicated by alizarin red S (ARS) staining, demonstrating positive effects of PIN on late osteoblast differentiation. In addition, PIN induced the mRNA level of BMP2, ALP, and osteocalcin (OCN). PIN also upregulated the protein level of BMP2 and increased canonical BMP2 signaling molecules, the phosphorylation of Smad1/5/8, and the protein level of Runt-related transcription factor 2 (RUNX2). Furthermore, PIN activated non-canonical BMP2 signaling molecules, activated MAP kinases, and increased β-catenin signaling. The findings of this study indicate that PIN has biological roles in osteoblast differentiation and matrix mineralization, and suggest that PIN might have anabolic effects in bone diseases such as osteoporosis and periodontitis.

## 1. Introduction

Osteoporosis is a systemic metabolic bone disease characterized by low bone mass and strength with fragility fractures and low-trauma fractures, which has recently become a major clinical problem [1]. It is also recognized as a multifactorial chronic systemic disease [2]. Bone fragility is caused by inadequate bone formation and excessive bone resorption, leading to decreased bone mass and micro architectural deterioration of bone tissue [1]. Osteoblasts, specialized cells derived from mesenchymal stem cells (MSCs), are essential for bone formation and maintenance, and osteoblast dysfunction causes pathological fragility and diseases such as osteoporosis and periodontitis [3]. Rovert et al. reported the anabolic effects of osteogenic compounds on human mesenchymal progenitor-derived osteoblasts and suggested the possibility of bone tissue engineering and bone diseases [4]. Anabolic treatments for osteoporosis increase the differentiation and maturation of osteoblast by altering bone metabolism [5,6]. However, safe and efficient drugs in improving osteoblast dysfunction and bone regeneration are limited, and it is much more difficult to treat bone diseases [3,6,7,8]. In recent years, there interest has grown in natural compounds to treat bone diseases due to low adverse effects and suitably long-term treatment when compared with chemically synthesized drugs [9,10]. Therefore, it is important to identify potential compounds to prevent and treat bone diseases.

*Styrax Japonica* Sieb. et Zucc. is a member of the Styracaceae family, which is a deciduous tree grown in the South Korea and Japan, including Central America, Mexico, and the Mediterranean region [11,12]. It has been used as traditional medicine in inflammatory diseases as well as in soap and cough medicine [13]. Previous studies on natural compounds isolated from the stem bark of *S. Japonica* demonstrated that norlignan and styraxlignolide significantly enhance anti-complement activity, Triterpenoid has biological effects via the expression of matrix metalloproteinases-1 and type 1 procollagen in skin diseases, and Styraxoside A has anti-inflammatory effects in RAW 264.7 Cell [14,15,16]. Pinoresinol glucoside (PIN) is isolated from the stem bark of *S. Japonica* belonging to lignans that have important pharmacological activities [11]. Previous phytochemical studies indicated that pinoresinol-4,4′-di-O-beta-D-glucoside from *Valeriana officinalis* induced calcium mobilization and cell migration through the activation of lysophosphatidic acid (LPA) receptor subtype in Mouse Embryo Fibroblasts [17]. Pinoresinol-4-*O*-*β*-D-glucoside isolated from *Artemisia selengensis* does not affect interleukin-6 (IL-6) production in TNF-α stimulated MG-63 cells [18]. However, effects of PIN isolated from the stem bark of *S. Japonica* in pre-osteoblast have not been defined yet.

In the present study, we investigate the underlying mechanism and biological effects of PIN on cell migration, osteoblast differentiation, and matrix mineralization in pre-osteblasts.

## 2. Results

### 2.1. PIN Increases Cell Migration during the Differentiation of Pre-Osteoblasts

**Figure 1:**
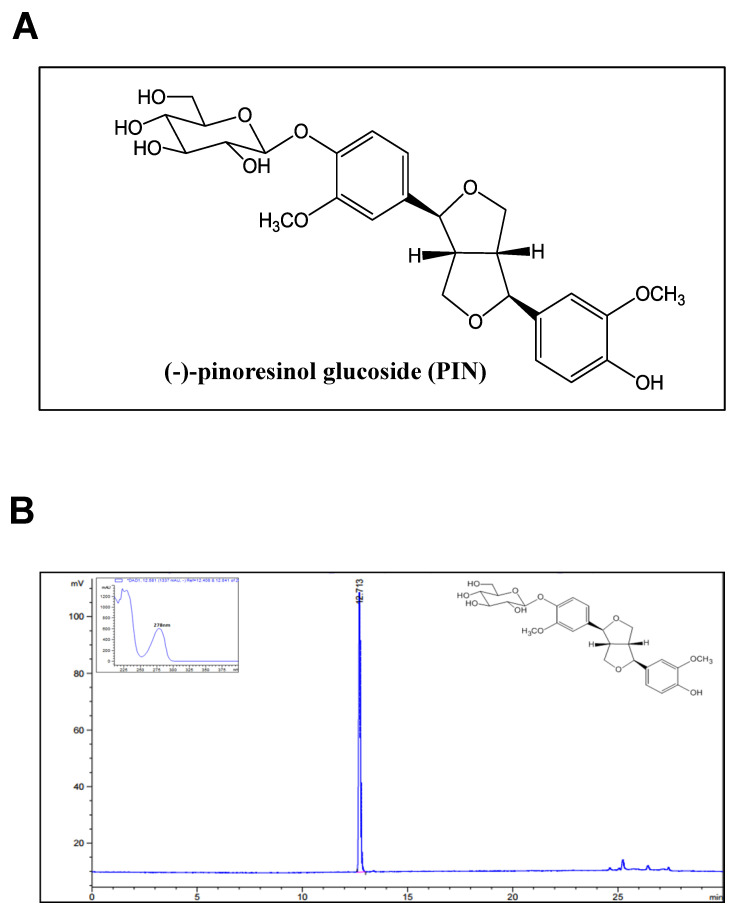
Chemical structures and HPLC chromatogram of pinoresinol glucoside (PIN). (**A**) Chemical structure of PIN from stem bark of *Styrax japonica*. (**B**) HPLC chromatogram of PIN.

**Figure 2:**
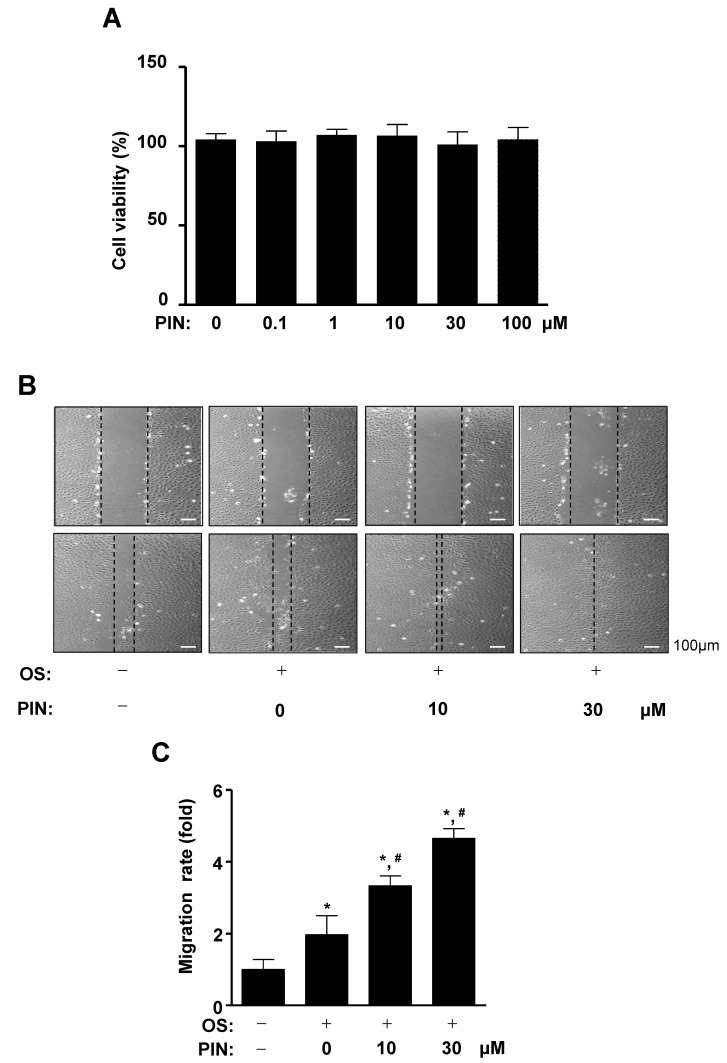
Effect of PIN on cytotoxicity in pre-osteoblasts and cell migration during osteoblast differentiation. (**A**) Pre-osteoblasts were cultured in 0.1, 1, 10, 30, and 100 µM of PIN f cell migration during osteoblast differentiation or 24 h. Cytotoxicity was assessed using an 3-[4,5-dimethylthiazol-2-yl]-2,5-diphenyltetrazolium bromide (MTT) assay and measured at a wavelength of 540 nm using a spectrophotometer. (**B**,**C**) After pre-osteoblasts were cultured in osteogenic supplement medium (OS) containing 50 μg/mL L-ascorbic acid (L-AA) and 10 mM β-glycerophosphate (β-GP) with 10 and 30 µM PIN for 24 h, cell migration was observed under a light microscope (**B**), and cell migration rate (fold) was measured and exhibited as a bar graph (**C**). Data represent the mean ± S.E.M. of experiments. *, *p* < 0.05 indicates statistically significant difference, compared with the control. #, *p* < 0.05 indicates statistically significant difference when compared with OS.

Pinoresinol glucoside (PIN) was isolated from the stem bark of *Styrax Japonica* Sieb. et Zucc (Figure 1A,B). To examine the effects of PIN on cell toxicity, pre-osteoblasts were treated with PIN for 24 h and cell viability was analyzed using an 3-[4,5-dimethylthiazol-2-yl]-2,5-diphenyltetrazolium bromide (MTT) assay. At concentrations ranging from 0.1 to 100 µM, PIN did not influence cell viability of pre-osteoblasts (Figure 2A). In order to examine whether PIN affects cell migration during the differentiation of pre-osteoblasts, we induced osteoblasts differentiation using osteogenic supplement medium (OS) containing 50 µg/mL l-ascorbic acid (L-AA) and 10 mM β-glycerophosphate (β-GP) in the absence and presence of 10 and 30 µM PIN for 24 h. The migratory effect of PIN was evaluated by wound healing assay, which revealed that PIN significantly promoted the closure rate of the cells in front of the wound area in a dose-dependent manner (Figure 2B,C).

### 2.2. PIN Increases the Early Differentiation of Pre-Osteoblasts

**Figure 3:**
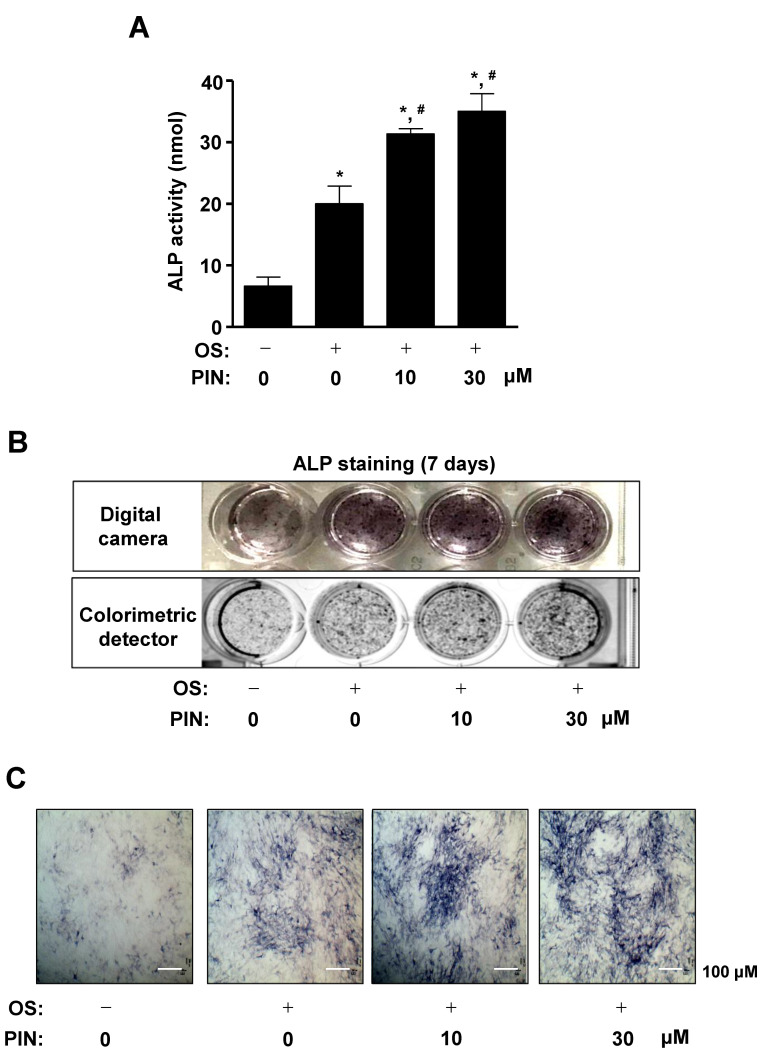
Effect of PIN on the staining and activity of ALP during the early differentiation of pre-osteoblasts. (**A**) Pre-osteoblasts were cultured in OS with 10 and 30 µM PIN for 7 days. The enzymatic activity of alkaline phosphatase (ALP) was measured at 405 nm using a spectrophotometer. (**B**,**C**) 7 days after osteoblast differentiation, ALP staining was detected by using a digital camera (upper) and colorimetric detector (bottom) (**B**), and ALP-positively-stained cells were also observed under a light microscope (**C**). Scale bar: 100 µm. Data represent the mean ± S.E.M. of experiments. *, *p* < 0.05 indicates statistically significant difference, compared with the control. #, *p* < 0.05 indicates statistically significant difference when compared with OS.

In order to investigate whether PIN affects osteoblast differentiation, 10 and 30 µM PIN were treated with OS for 7 days, and alkaline phosphatase (ALP) enzymatic activity was measured to detect the early differentiation of pre-osteoblasts. We found that ALP activity was significantly increased in response to treatment with PIN in a dose-dependent manner during osteoblast differentiation (Figure 3A). Consistent with the assay results, ALP staining was observed a digital camera and colorimetric detector, which showed that PIN treatment promoted the osteoblast differentiation (Figure 3B). Using a light microscope, the effects of PIN on osteoblast differentiation were also confirmed on light microscopy observations (Figure 3C).

### 2.3. PIN Increases Mineralized Nodule Formation during Differentiation of Pre-Osteoblasts

**Figure 4:**
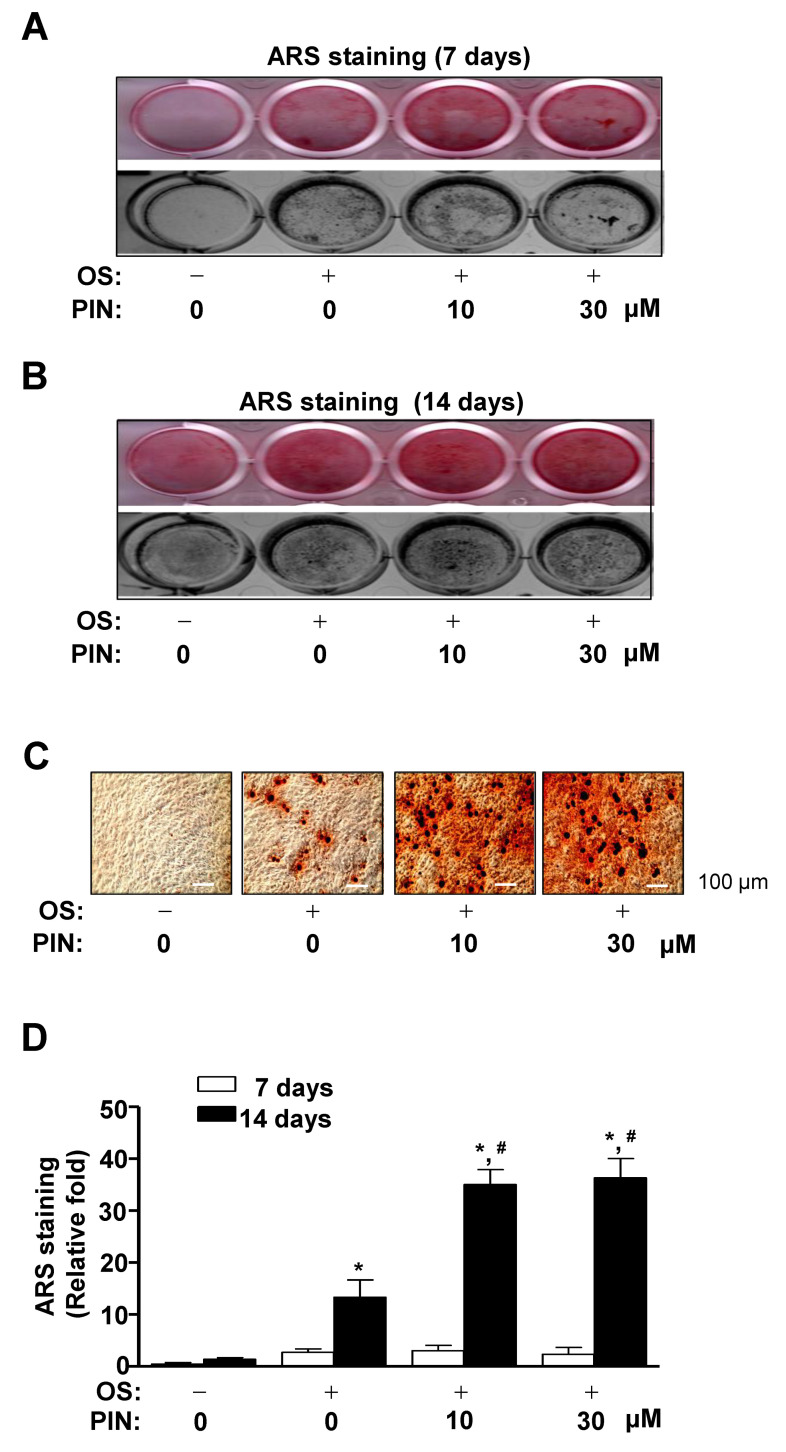
Effect of PIN on mineralized nodule formation during the late differentiation of pre-osteoblasts (**A**–**D**). Pre-osteoblasts were cultured in OS with 10 and 30 µM PIN, and mineralized nodule formation was performed using the Alizarin red S (ARS) staining assay at 7 (**A**) and 14 days (**B**). The ARS staining was detected by using a scanner (upper) and colorimetric detector (bottom) (**A**,**B**). Mineralized nodule formation was observed under a light microscope (**C**) and quantified by measuring at a wavelength of 590 nm using a spectrophotometer (**D**). Scale bar: 100 µm. Data represent the mean ± S.E.M. of experiments. *, *p* < 0.05 indicates statistically significant difference, compared with the control. #, *p* < 0.05 indicates statistically significant difference when compared with OS.

We subsequently investigated whether PIN influences the late differentiation of pre-osteoblasts based on the degree of matrix mineralization using Alizarin red S (ARS) staining. After we induced osteoblasts differentiation using OS in the absence and presence of PIN (10 and 30 µM), we observed the formation of mineralized nodule at 7 and 14 days using a scanner and colorimetric detector. The matrix mineralization was formed at 14 days, which revealed that PIN treatment promoted the late osteoblasts differentiation of pre-osteoblasts in a dose-dependent manner (Figure 4A,B). Using a light microscope, PIN-induced mineralized nodule formation was also visualized (Figure 4C). Consistent with the observations, effects of PIN on late osteoblast differentiation were statistically validated by the quantification of ARS staining (Figure 4D).

### 2.4. PIN Increases BMP2 Expression and Activates Its Signaling during Differentiation of Pre-Osteoblasts

**Figure 5:**
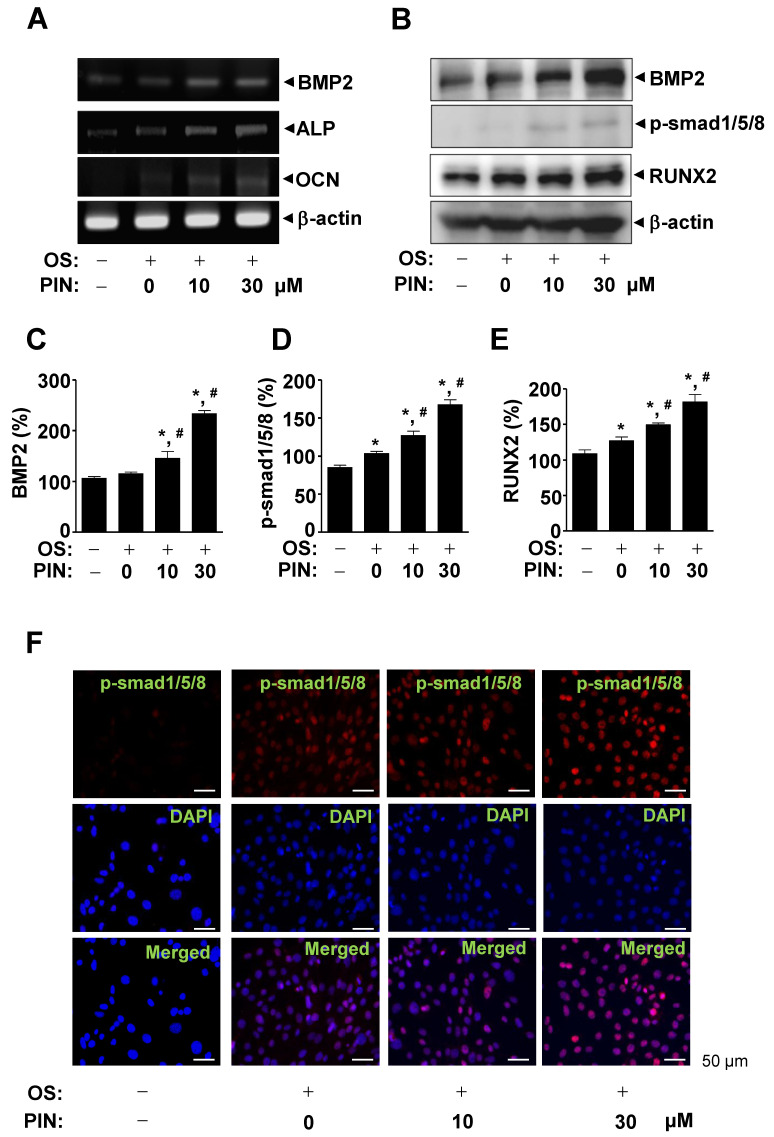
Effect of PIN on BMP2 signaling and gene expression during osteoblast differentiation. (**A**) After 10 and 30 µM PIN was treated for 3 days in osteoblast differentiation, total RNA was isolated. The mRNA levels of BMP2, ALP, OCN, and β-actin were analyzed by using RT-PCR. β-actin was used as a loading control. (**B**–**E**) After 24 h, the protein levels of BMP2, p-Smad1/5/8, RUNX2, and β-actin were assessed using western blot analysis. β-actin was used as a loading control (B). The levels of BMP2 (**C**), p-Smad1/5/8 (**D**), and RUNX2 (**E**) were represented as relative percentages of the control and exhibited as a bar graph. (**F**) After 24 h, p-smad1/5/8 was immunostained with rabbit anti-p-smad1/5/8 antibody, followed by Alexa-Fluor 568-conjugated secondary antibody (red) and counterstained with DAPI (blue). The bottom panels show the merged images of the up and middle panels. Scale bar: 50 µm Data represent the mean ± S.E.M. of experiments. *, *p* < 0.05 indicates statistically significant difference, compared with the control. #, *p* < 0.05 indicates statistically significant difference when compared with OS.

To further investigate the molecular mechanisms underlying the effects of PIN on the differentiation of pre-osteoblasts, we examined bone morphogenetic protein (BMP)-Smad1/5/8-Runt-related transcription factor 2 (RUNX2) signaling using RT-PCR and western blotting. Ten and 30 µM PIN upregulated the mRNA level of BMP2 and its target osteoblast genes, ALP and osteocalcin (OCN) (Figure 5A). PIN also enhanced protein level of BMP2, followed by the phosphorylation of Smad1/5/8 and the expression of RUNX2 (a key transcription factor in osteoblasts (Figure 5B–E)). Immunofluorescence observation using confocal microscopy also confirmed an increase in the phosphorylation of Smad1/5/8 in response to PIN treatment (Figure 5F).

### 2.5. PIN Increases Non-Canonical BMP2 Signaling and β-Catenin Sigaling during Differentiation of Pre-Osteoblasts

**Figure 6:**
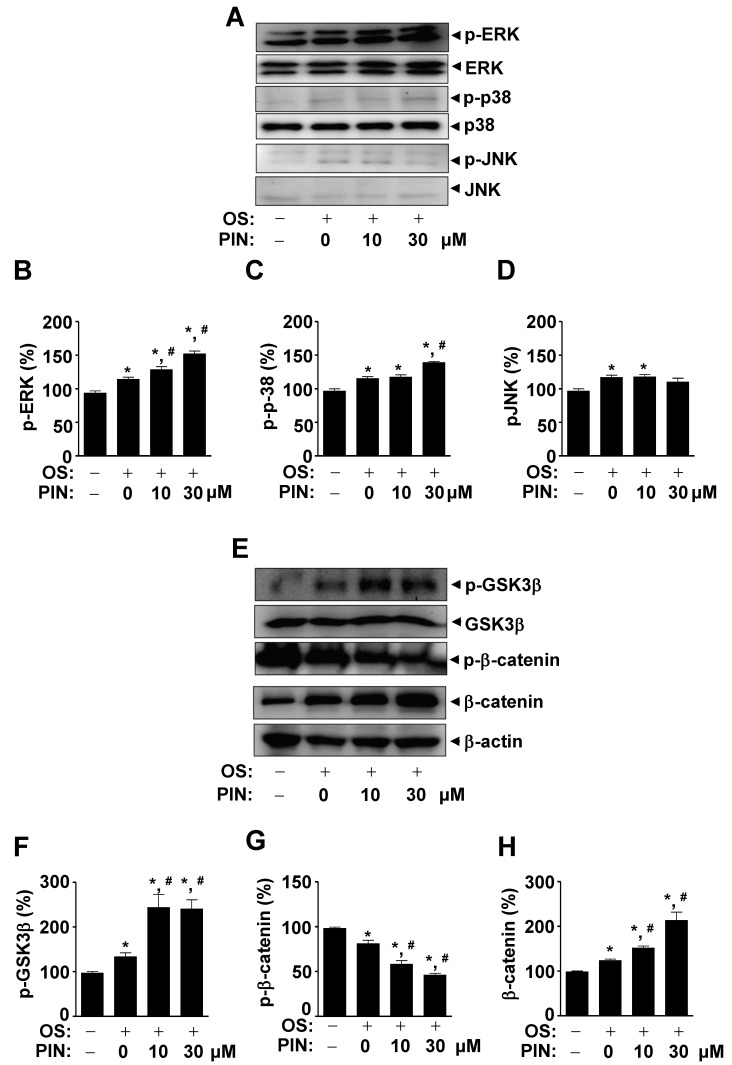
Effect of PIN on ERK, JNK, p38, and β-catenin signals during osteoblast differentiation. (**A**–**G**) After pre-osteoblasts were differentiated with 0 and 30 µM PIN for 24 h, Phospho (p)-ERK, ERK2, p-p38, p38, p-JNK, and JNK (**A**), and p-GSK3β, GSK3β, p-β-catenin, β-catenin, and β-actin (E) were analyzed using western blot analysis. The levels of p-ERK (**B**), p-p38 (**C**), p-JNK (**D**), p-GSK3β (**F**), p-β-catenin (**G**), and β-catenin (**H**) were represented as relative percentages of the control and are exhibited as a bar graph. Data represent the mean ± S.E.M. of experiments. *, *p* < 0.05 indicates statistically significant difference, compared with the control. #, *p* < 0.05 indicates statistically significant difference when compared with OS.

We subsequently investigated whether PIN influences non-canonical BMP2 signaling using western blotting. We found that 10 and 30 µM PIN increased the activities of the non-canonical BMP2-mediated MAPKs pathways, including ERK1/2 and p38 (Figure 6A–D). Under same condition, we also investigated β-catenin signaling since β-catenin also plays an important role in osteoblast differentiation and bone formation. These resulted in the absence and presence of 10 and 30 µM PIN during osteoblast differentiation, and revealed that PIN increased in the phosphorylation of GSK3β and the protein level of β-catenin in response to the treatment of PIN (Figure 6E–H). We also validated that PIN-induced osteoblast differentiation is mediated by BMP2 and β-catenin signaling (Supplementary Figure S1).

## 3. Discussion

Bone formation contains complex process, including the proliferation of osteoblast lineage cells, the mobilization and differentiation of pre-osteoblasts, and the maturation of osteoblasts [9]. After bone destruction, the recovery process of bone tissues, including cell migration, differentiation, and bone matrix mineralization, is also dependent on osteoblasts [19,20]. Thus, osteoblasts regulate bone homeostasis from birth until death, and the dysregulation of osteoblasts causes etiology of bone diseases such as osteoporosis [9,21]. In the present study, we found that PIN increased the cell migration of osteoblasts. The migration of osteoblasts from the bone marrow, periosteum, surrounding tissues, and circulating blood is required to form bone tissue through the synthesis, secretion, and mineralization of the bone matrix [19,22,23]. We also demonstrated that PIN promotes the expression and enzymatic activity of ALP and the formation of mineralized nodules. It is well known that ALP activity is an early marker of osteoblast differentiation, and when mature osteoblasts form matrix mineralization by calcium deposition it is a late and mature marker of osteoblast differentiation [21,24,25]. Based on the literature and our results, the findings of this study suggest that PIN is responsible for bone formation and bone homeostasis through cell migration, differentiation, and mineralization in osteoblasts.

A complex network activated by specific osteogenic signals controlled osteoblast differentiation [26,27]. In the present study, we demonstrated that PIN increased the expression of BMP2, the phosphorylation of Smad1/5/8, and the expression of RUNX2. BMP2, an osteogenic factor, activates the Smad1/5/8 and RUNX2 pathways to produce bone-specific proteins, including osterix, collagen type I, ALP, and OCN through RUNX2, a key transcriptional factor in osteoblast differentiation [28,29,30,31]. We also investigated BMP2 signaling target genes, and our results demonstrated that PIN increases the mRNA levels of ALP and OCN on osteoblast differentiation. These findings indicate that PIN increases osteoblast differentiation by regulating the BMP2 signaling pathway.

BMP2 signaling also activates the non-canonical pathway via MAPKs, including ERK1/2, p38, and JNK1/2, to control cell migration and differentiation in osteoblasts [32,33,34,35]. In the present study, we found that PIN activates ERK1/2 and p38 MAPKs during the differentiation of pre-osteoblasts. Previous studies have reported that MAPKs signaling pathways are also involved in Wnt/β-catenin signaling by regulating β-catenin levels and β-catenin-induced gene transcription [36]. Wnt/β-catenin signaling plays a critical function in bone development, formation, and maintenance [9,37]. In addition, there is functional cross-talk between BMP2 and Wnt/β-catenin signals in osteoblast differentiation and bone homeostasis, including the fact that Wnt/β-catenin signaling affects BMP2 expression and BMP2-mediated osteoblast-specific gene expression in osteoblasts, and vice versa [38,39,40,41,42]. We also demonstrated that PIN increased GSK3β phosphorylation and β-catenin levels in osteoblasts. Thus, our results suggest that PIN has anabolic effects in osteoblast differentiation and matrix mineralization by regulating BMP2 and Wnt/β-catenin signaling.

In conclusion, our results first demonstrated that PIN isolated from isolated from the stem bark of *S. Japonica* has biological effects on cell migration, differentiation, and mineralization through BMP2 and β-catenin signals in osteoblasts. Plant-derived natural compounds are becoming increasingly relevant in the treatment and prevention of bone diseases because natural compounds have been used to treat many diseases and have shown fewer adverse reactions than chemically synthesized medicines [9,43,44]. Therefore, the findings of this study suggest that PIN might be a potential source in the development of anabolic drugs to prevent and treat bone diseases such as osteoporosis and periodontal disease.

## 4. Materials and Methods

### 4.1. General

The methanol (MeOH), *n*-hexane (Hx), ethyl acetate (EtOAc), chloroform (CHCl_3_), and acetonitrile (ACN) were purchased from Duksan Chemical Co. (Seoul, Korea). The column chromatography used silica gel 60 (Merck 230–400 mesh, ASTM, Darmstadt, Germany) and ODS-A (Merck ASTM, Darmstadt, Germany). The NMR spectra were recorded on a JEOL ECX-500 spectrometer, operating at 500 MHz for ^1^H and 125 MHz for ^13^C NMR spectrum (JEOL Ltd., Tokyo, Japan).

### 4.2. Plant Material

The stem bark of *Styrax Japonica* Sieb. et Zucc. was collected at Jeju (Korea) in July 2002. A voucher specimen (00250) was verified by Dr. Tae-Jin Kim, Korea Research Institute of Bioscience and Biotechnology (KRIBB), Korea.

### 4.3. Extraction and Isolation of Compound

The dried stem bark of *Styrax Japonica* Sieb. et Zucc. (12.0 kg) was extracted with MeOH. The MeOH extract (1.4 kg) was suspended with 2000 mL of distilled water and solvent partitioned using same volume of Hx and EtOAc. The EtOAc soluble fraction (78.0 g) was separated into five fractions (SJE 1~SJE 5) by chromatography on a silica gel column eluted with a gradient of CHCl_3_ and MeOH (10:1 to 1:1). The fraction SJE 4 (19.3 g) was re-chromatographed using ODS-A column with 25% aqueous ACN to collect five fractions (SJE 4-1~SJE 4-5). The subfraction SJE 4-2 (8.2 g) was purified by medium pressure liquid chromatography (MPLC) on a Lichroprep RP-18 (25–40 μm, Merck, Darmstadt, Germany) eluted with 30% aqueous ACN to obtain active compound (1.3 g, 98.7% purity). The purity of active compound was established by HPLC. The chemical structure of active compound was determined on the basis of spectroscopic data and comparison of those before literatures and identified as a PIN [45].

### 4.4. PIN

White powder; ESI-MS *m*/*z* = 520.5 [M]^+^, molecular formula C_26_H_32_O_11_; ^1^H NMR (500 MHz, Pyridine-*d*_5_) *δ* 7.57 (1H, d, *J*=8.4 Hz, H-5), 7.23 (3H, m, H-5′, 6, 6′), 7.06 (1H, d, *J*=1.9 Hz, H-2′), 6.94 (1H, d, *J*=1.9 Hz, H-2), 4.62 (1H, d, *J*=7.4 Hz, Glc-1), 4.32 (7H, m, H-7′, Glc-2_~_Glc-6), 4.11 (1H, m, H-7), 3.97 (2H, m, H-9eq and H-9′eq), 3.76, 3.73 (each 3H, s, OCH_3_), 3.57 (2H, m, H-9ax and H-9′ax), 3.15 (2H, m, H-8 and H-8′); ^13^C-NMR (125 MHz, Pyridine-*d*_5_) *δ* 150.2 (C-4′), 148.0 (C-3), 146.8 (C-4), 146.6 (C-3′), 137.1 (C-1), 133.7 (C-1′), 119.3 (C-6), 118.9 (C-6′), 117.3 (C-5), 115.3 (C-5′), 111.2 (C-2), 110.4 (C-2′), 102.3 (Glc-1), 86.4 (C-7), 86.2 (C-7′), 77.7 (Glc-3), 77.6 (Glc-5), 74.5 (Glc-2), 72.2 (C-9), 72.1 (C-9′), 71.1 (Glc-4), 62.5 (Glc-6), 56.3 (OCH_3_), 56.1 (OCH_3_), 55.2 (C-8), and 55.1 (C-8′). PIN (0.1, 1, 10, 30, and 100 µM) was dissolved in 100% DMSO, diluted 1:1000 in final concentration, and 0.1% DMSO was used as the vehicle control.

### 4.5. Cell Culture

MC3T3-E1 pre-osteoblasts (#CRL-2593) purchased from the American Type Culture Collection (ATCC) (Manassas, VA) were kindly provided by the Bioevaluation Center (Korea Research Institute of Bioscience and Biotechnology, Korea). The cells were cultured in *α*-minimum essential medium (*α*-MEM) without L-ascorbic acid (WELGEME, Inc., Seoul, Korea) supplemented with 10% fetal bovine serum (FBS), penicillin (100 units/mL), and streptomycin (100 μg/mL) at 37 °C in a humidified atmosphere of 5% CO_2_ and 95% air.

### 4.6. Osteoblast Differentiation

Osteoblast differentiation was induced using osteogenic supplement medium (OS) containing 50 µg/mL L-ascorbic acid (L-AA) and 10 mM β-glycerophosphate (β-GP) (Sigma-Aldrich, St. Louis, MO, USA). PIN (10 and 30 µM) was dissolved in 100% DMSO, diluted 1:1000 in final concentration, and 0.1% DMSO was used as the vehicle control. The medium was replaced every 2 days during the incubation period [46].

### 4.7. MTT Assay

Cell viability was detected using MTT (Sigma-Aldrich) solution [47]. Absorbance was measured at a wavelength of 540 nm using the Multiskan GO Microplate Spectrophotometer (Thermo Fisher Scientific, Waltham, MA, USA).

### 4.8. Western Blot Analysis

Western blot analysis was carried out as previously described [48]. Briefly, equal amounts of lysate (20 μg) were resolved by sodium dodecyl sulfate-polyacrylamide gel electrophoresis (SDS-PAGE) and transferred to a polyvinylidene fluoride (PVDF) membrane (Millipore, Bedford, MA, USA). The membrane was blocked for 1 h at room temperature and incubated overnight at 4 °C with the specific primary antibodies as follows: RUNX2 (O1L7F; 1:1000,#12556S, Cell Signaling Cell Signaling Technology, Beverly, MA, USA), p-Smad1/5/8 (1:2000, #13820S, Cell Signaling), USA), β−actin (1:1000, #sc-47778, Santa Cruz Biotechnology), p-ERK1/2 (1:2000, #9101S, Cell Signaling Technology), ERK1/2 (1:2000, #9102, Cell Signaling), p-p38 (1:1000, #9211, Cell Signaling), p38 (1:1000, #9212, Cell Signaling), p-JNK (1:1000, #9251, Cell Signaling), JNK (1:1000, #9252, Cell Signaling), GSK3β (1:1000, #12456, Cell Signaling), p-GSK3β (1:1000, #9336, Cell Signaling), and β-catenin (1:1000, #8480, Cell Signaling). After washing, the membrane incubated with diluted horseradish peroxidase (HRP)-conjugated secondary antibodies (1:10,000, Jackson ImmunoResearch, West Grove, PA, USA) for 2 h at room temperature and detected with Immobilon Western Chemiluminescent HRP Substrate (Merck, Darmstadt, Germany) using the ProteinSimple detection system (ProteinSimple Inc., Santa Clara, CA, USA).

### 4.9. Cell Migration Assay

Cell migration was accessed using an in vitro wound healing assay [46]. Briefly, the cells were wound with a 200 μL pipette tip and cultured for 24 h at 37 °C in a humidified atmosphere of 5% CO_2_ and 95% air. Cell migration was observed using a light microscope, and the migration images were compared to quantify cell migration rate.

### 4.10. Alkaline Phosphatase (ALP) Activity Assay

Osteoblast differentiation was induced using OS containing 50 μg/mL L-AA and 10 mM β-GP with PIN (10 and 30 μM) for 7 days. The cell lysates were performed according to the manufacturer’s protocol using alkaline phosphatase activity colorimetric assay kit (Biovision, Milpitas, CA, USA) [46].

### 4.11. ALP Staining Assay

Osteoblast differentiation was induced using OS containing 50 μg/mL L-AA and 10 mM β-GP with PIN (10 and 30 μM) for 7 days. Cells were washed with 1 × PBS and then fixed in 10% formalin for 10 min at room temperature. After washing with distilled water, the cells were incubated with substrate solution for the reaction of ALP, followed according to the manufacturer’s protocol (Takara Bio Inc., Tokyo, Japan) as previously described [46].

### 4.12. Alizarin Red S (ARS) Staining

Osteoblast differentiation was induced using OS containing 50 μg/mL L-AA and 10 mM β-GP with PIN (10 and 30 μM) for 7 and 14 days. Cells were fixed in 10% formalin for 10 min at room temperature, washed with distilled water, and were stained with 2% ARS (pH 4.2) (Sigma-Aldrich) for 10 min. The level of ARS staining was observed using a scanner and colorimetric detector (ProteinSimple Inc., Santa Clara, CA, USA). To quantify and validate, stains were dissolved using 100% DMSO and measured at a wavelength of 590 nm using the Multiskan GO Microplate Spectrophotometer (Thermo Fisher Scientific).

### 4.13. Immunofluorescence

Immunofluorescence was performed [49]. Briefly, the cells were fixed, permeabilized, blocked, and incubated overnight at 4 °C with the specific primary antibodies. After washing, the cells were incubated with an anti-rabbit secondary antibody labeled with Alexa-Fluor 568 (1:500 dilution, Invitrogen, Carlsbad, CA, USA) for 2 h at room temperature in the dark and counterstained with 4′,6-diamidino-2-phenylindole (DAPI) (Sigma-Aldrich) for 10 min at room temperature in the dark. The cells were washed three times, mounted on glass slides, and observed using a confocal microscope (K1-Fluo Confocal Laser Scanning Microscope, Korea).

### 4.14. Reverse Transcription-Polymerase Chain Reaction (RT-PCR)

Total RNA from cells was extracted using TRIzol™ reagent (Life Technologies, Gaithersburg, MD) according to the manufacturer’s instructions. RNA (1 µg) isolated from each sample was reverse-transcribed using oligo (dT)_15_ primers with AccuPower^®^ CycleScript RT PreMix (Bioneer Corporation, Daejeon, Korea). Next, the resultant cDNA was amplified with AccuPower^®^ PCR PreMix (Bioneer Corporation). The sequences of primers were as follows:

BMP2, F: 5′-ACACAGCTGGTCACAGATAAG-3′, R: 5′-CTTCCGCTGTTTGTGTTTGG-3′; ALP, F: 5′-ACACCTTGACTGTGGTTACTG-3′, R: 5′-CCATATAGGATGGCCGTGAAG-3′; OCN, F: 5′-ACACCATGAGGACCATCTTTC-3′, R: 5′-CGGAGTCTGTTCACTACCTTATT-3′; β-actin, F: 5′-AATGTGGCTGAGGACTTTG-3′, R: 5′-GGGACTTCCTGTAACCACTTATT-3′.

### 4.15. Statistical Analysis

The data were analyzed using Prism Version 5 program (GraphPad Software, Inc., San Diego, CA, USA). All numeric values are presented as the means ± S.E.M. The statistical significance of data was determined using a Student’s unpaired *t* test. A value of *p* < 0.05 was considered to indicate statistical significance.

## Supplementary Materials

Supplementary materials can be found at https://www.mdpi.com/1422-0067/21/24/9579/s1.
