# Supplementary material for: Effects of PIN on Osteoblast Differentiation and Matrix Mineralization through Runt-Related Transcription Factor
Source: Int J Mol Sci. 2020 Dec 16;21(24):9579. doi: 10.3390/ijms21249579 (PMC7765567; doi:10.3390/ijms21249579)
Supplement: Supplementary file 1 [file ijms-21-09579-s001.pdf]

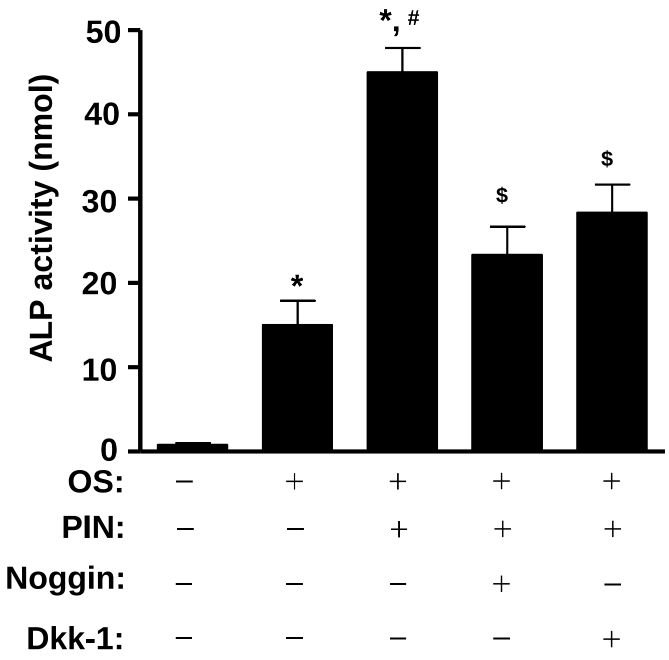

Supplementary Figure 1. PIN-induced osteoblast differentiation is mediated by BMP2 and β-catenin signaling. After pre-osteoblasts were differentiated with PIN in the absence and presence of Noggin and Dkk-1 for 7 days, ALP activity was measured at 405 nm using a spectrophotometer

Data represent the mean ± S.E.M. of experiments. *: statistically significant difference compared with the control ($p < 0.05$). #: statistically significant difference compared with OS ($p < 0.05$).). $: statistically significant difference compared with the OS + PIN ($p < 0.05$).
